# Paroxysmal Genetic Movement Disorders and Epilepsy

**DOI:** 10.3389/fneur.2021.648031

**Published:** 2021-03-23

**Authors:** Claudio M. de Gusmão, Lucas Garcia, Mohamad A. Mikati, Samantha Su, Laura Silveira-Moriyama

**Affiliations:** ^1^Department of Neurology, Boston Children's Hospital and Harvard Medical School, Boston, MA, United States; ^2^Department of Neurology, Universidade Estadual de Campinas (UNICAMP), São Paulo, Brazil; ^3^Department of Medicine, Universidade 9 de Julho, São Paulo, Brazil; ^4^Division of Pediatric Neurology and Developmental Medicine, Duke University Medical Center, Durham, NC, United States; ^5^Education Unit, University College London Institute of Neurology, University College London, London, United Kingdom

**Keywords:** paroxysmal kinesigenic dyskinesia, paroxysmal non-kinesigenic dyskinesia, paroxysmal exercise induced dyskinesia, episodic ataxia, infantile convulsions and choreoathetosis syndrome, generalized epilepsy and paroxysmal dyskinesia

## Abstract

Paroxysmal movement disorders include paroxysmal kinesigenic dyskinesia, paroxysmal non-kinesigenic dyskinesia, paroxysmal exercise-induced dyskinesia, and episodic ataxias. In recent years, there has been renewed interest and recognition of these disorders and their intersection with epilepsy, at the molecular and pathophysiological levels. In this review, we discuss how these distinct phenotypes were constructed from a historical perspective and discuss how they are currently coalescing into established genetic etiologies with extensive pleiotropy, emphasizing clinical phenotyping important for diagnosis and for interpreting results from genetic testing. We discuss insights on the pathophysiology of select disorders and describe shared mechanisms that overlap treatment principles in some of these disorders. In the near future, it is likely that a growing number of genes will be described associating movement disorders and epilepsy, in parallel with improved understanding of disease mechanisms leading to more effective treatments.

## Introduction

The concept of neurological diseases with episodic manifestations is not foreign to clinical practice. Neurologists frequently encounter patients with “attacks” with minimal or no abnormalities on the examination in-between spells. Initially, neurological nosology relied on patient history or witnessed account from bystanders. This led to using descriptive but non-specific terms to classify disease, such as “faints,” “apoplexy,” or “convulsions.” Over time, improved understanding of the underlying physiological abnormality (or at least refinement of semiology) led to more precise nomenclature. For example, migrainous auras are characterized by transient neurologic deficits associated with cortical spreading depression while epilepsy is caused by synchronous abnormal firing of a group of cortical neurons. Altered perfusion with transient lack of oxygen supply to any central nervous system structure characterizes a transient ischemic attack. Notwithstanding nebulous pathophysiology, transient motoric phenomena attributed to abnormal basal ganglia and/or cerebellar activity are therefore labeled paroxysmal movement disorders (encompassing paroxysmal dyskinesia and episodic ataxia).

Although large efforts have been made to discriminate paroxysmal movement disorders from epileptic seizures, recent progress in neurogenetics has pointed to growing intersections between genotypes, phenotypes, and treatment in several conditions with paroxysmal neurological symptoms. In this review, we aim to revisit this relationship showing how certain gene mutations can manifest with paroxysmal dyskinesia, episodic ataxia and/or epilepsy (Table 1). Furthermore, we consider how treatment for these conditions overlaps considerably.

**Table 1:** Phenotypic clues in select neurogenetic syndromes associated with paroxysmal neurological symptoms [modified from Silveira-Moriyama et al. (1)].

**Gene**	**Paroxysmal dyskinesia**	**Episodic ataxia**	**Epilepsy**	**Inheritance**	**Phenotype summary**
*PRRT2*	✓	✓	✓	AD	**Most commonly:** Classic PKD, benign familial infantile seizures, hemiplegic migraine, episodic ataxia. PKD has short duration (<1 min), high frequency (>daily), often asymmetric or unilateral, but also bilateral; may be chorea/dystonia/mix; may present sensory aura before attack; excellent response to low dose antiepileptic (carbamazepine and phenytoin are the most used, in this order); onset in the second decade of life and improvement or remission in the fourth decade of life. Family history of epilepsy or migraine is common.
*SCN8A*	✓		✓	AD	Most commonly developmental epileptic encephalopathy with onset < 2 years old; interictal movement disorders, tremor and hyperekplexia. Milder phenotypes exist combining BFIS, PKD.
*DEPDC5*	✓		✓	AD	**Most commonly:** Focal family epilepsy with variable foci. Has been reported in the “typical” PKD phenotype, with abnormal interictal EEG.
*CHRNA4*	✓		✓	AD	**Most commonly:** Autosomal dominant nocturnal frontal lobe epilepsy. Anecdotal reports of individuals with paroxysmal dyskinesia
*SLC16A2*	✓			XL	Allan Herndon-Dudley syndrome: Intellectual disability, choreoathetosis, spastic paraparesis, and thyroid hormone abnormalities. May have associated paroxysmal dyskinesia sometimes triggered by passive movements.
*PNKD*	✓			AD	**Most common phenotype:** PNKD starting in infancy or childhood; mix of chorea and dystonia; attacks lasting between min and 1 h, not triggered by sudden movement or exercise, but triggered by caffeine or alcohol intake, as well as emotional stress; response to benzodiazepines; not associated with epilepsy. May have migraine.
*ATP1A3*	✓	✓	✓	AD	**Most common phenotypes: AHC, RDP, CAPOS, D-DEM**Ø. AHC starts in infancy and presents with episodes of hemiplegia and often hemidystonia. It usually evolves with epilepsy and loss of developmental milestones, and often persistent movement disorder. The RDP phenotype does not usually cause paroxysmal dystonia, but persistent dystonia and/or parkinsonism of subacute onset. Paroxysmal ataxia, later becoming persistent, is also described in *ATP1A3* mutations. Epilepsy is most consistently associated with AHC and has infantile onset in most.
*ATP1A2*	✓		✓	AD	Alternating hemiplegia of childhood; Hemiplegic migraine. Some cases described with epilepsyand exacerbations including encephalopathy and paroxysmal dystonia.
*KCNMA1*	✓		✓	AD, AR^*^	In AD state, may resemble “classic” PNKD, but with associated epilepsy and developmental delay. May respond to stimulant. Phenotype is more severe in AR mutations with epilepsy, intellectual disability and cerebellar atrophy.
*SLC2A1*	✓	✓	✓	AD, AR^*^	**Most common phenotypes**: Infantile epilepsy, classic PED, PED associated with epilepsy. PED often in lower limbs; dystonia>chorea; low glucose CSF in some but not all patients; usually does not respond well to antiepileptic drugs. Age at onset is quite variable from childhood to adulthood. Family history of epilepsy is common. Variable penetrance and expressivity. Very rare homozyogous/compound heterozygous cases.
*ECHS1*	✓		✓	AR	Leigh-disease like phenotype with clinical and imaging characteristics. Some patients may have paroxysmal exertional dyskinesia, either as an isolated finding or as an intermediate phenotype.
*TBC1D24*	✓	✓	✓	AR	Expanding phenotype including developmental delay, hearing impairment, DOORS syndrome, epilepsy, myoclonus, and paroxysmal neurological symptoms including PKD, PED and EA
*GCH1*	✓			AD, AR^*^	**Most common phenotypes:** Dopa responsive dystonia. PED with dystonic features is a rare initial presentation in children, usually in the lower limbs, with excellent response to levodopa; *GCH1* has AD inheritance but recessive forms are possible with usually with more severe phenotype and infantile onset.
*PARKIN*	✓			AR	**Most common phenotypes: levodopa responsive Parkinsonism**. PED with dystonic features is rarely seen, usually in the lower limbs, with excellent response to levodopa, usually starts in young adulthood. Family history of consanguinity can be a clue since AR inheritance.
*ACDY5*	✓			AD	**Most common phenotypes:** Familial dyskinesia with facial myokymia. Paroxysmal dyskinesia brought on by stress or sleep may be the initial presentation, some patients have history of developmental delay or hypotonia. Older family members may present with persistent dyskinesias; dyskinesias may also improve in adult life.
*KCNA1*	✓	✓	✓	AD	**Most common phenotype:** Episodic ataxia with interictal myokymia. A subset may have epilepsy with partial or generalized seizures, rarely epileptic encephalopathy.
*CACNA1A*		✓	✓	AD	**Most common phenotype:** Episodic ataxia with interictal nystagmus; some may have cerebellar atrophy and progressive interictal ataxia. Can respond to acetazolamide. Other paroxysmal disorders include hemiplegic migraine, epilepsy and possibly paroxysmal torticollis and paroxysmal tonic upgaze. Emerging evidence of role in neurodevelopmental disabilities.
*CACNB4*		✓	✓	AD, AR^*^	AD inheritance with episodic ataxia, predisposition to epilepsy. In recessive state can have severe phenotype.
*SLC1A3*		✓	✓	AD	Phenotype similar to *CACNA1A* (EA2), some reported cases with seizures, migraine, alternating hemiplegia and chronic ataxia.
*SCN2A*		✓	✓	AD	Wide phenotypic variability with epileptic encephalopathy, benign familial neonatal seizures, interictal hyperkinetic movement disorder, and episodic ataxia.
*KCNA2*		✓	✓	AD	Epileptic encephalopathy and chronic ataxia, some patients reported episodic attacksa and infantile-onset seizures

*AD, Autosomal Dominant; AR, Autosomal Recessive: XL, X-Linked, DOORS, Deafness, Onychodystrophy, Osteodystrophy, Mental Retardation Syndrome; EA, Episodic Ataxia*.

The ^*^ indicates a possible but less common mode of inheritance.

## Historical Overview and Underlying Genetic Conditions

The first description of paroxysmal dyskinesia is attributed to Kure, who made use of photography in the nineteenth century to document what was likely paroxysmal kinesigenic dyskinesia (PKD) (2, 3). Similar cases were described by Gowers in his seminal book on Epilepsy (4). For a period of time such spells were believed to be seizures, illustrating the reasoning why antiepileptic drugs were used for treatment of such spells (5, 6). To this day, some authors paraphrase Gowers by including these disorders on the borderland of epilepsy (7). The initial clinical classifications for paroxysmal dyskinesia attempted to classify the phenotype by motor phenomenology, using terms like “paroxysmal dystonic choreoathetosis” or “paroxysmal kinesigenic choreoathetosis” (6, 8). Subsequently, Demirkiran and Jankovic proposed a classification scheme that divided the paroxysmal dyskinesias highlighting the type of trigger as the main phenotypic characteristic, creating the kinesigenic (PKD), non-kinesigenic (PNKD), exertional (PED), and hypnogenic (PHD) subtypes (9). This classification proved useful and gained widespread acceptance; it has been the framework toward which phenotypes have been organized, leading to the unraveling of their genetic underpinnings (10). In 2004 mutations in the *PNKD* gene (previously called *MR-1*) were associated with PNKD (11). In 2008, mutations in the facilitated glucose transporter 1 (previously called *GLUT1*, now named *SLC2A1* gene) were found in patients with PED and in 2011, mutations in *PRRT2* were associated with PKD (12–14). Since its description, most cases of PHD were found to be largely attributed to nocturnal frontal lobe seizures, with rare isolated cases reported in association with *PRRT2* (15–17).

The episodic ataxia phenotype was first described by Parker in 1946, who used the term “periodic” to describe a syndrome of intermittent ataxia with familial clustering and the potential for developing progressive ataxia (18, 19). Awareness of the condition increased in the ensuing decades, with descriptions of autosomal dominant inheritance and the serendipitous discovery that many patients responded favorably to acetazolamide (20–24). The clinical classification of episodic ataxia is based on presence of interictal symptoms, triggers, spell characteristics, and response to treatment. Nevertheless, they have been organized according to the suspected or known locus in the order they were described, giving rise to the terminology still used to this day (EA1, EA2, etc.) (25). Similarly to the paroxysmal dyskinesias, the clinical description has also led to the discovery of underlying genetic defects. Thus, starting in 1994, episodic ataxia type 1 (EA1) was associated with mutations in the gene *KCNA1* which codes for one of the units of the potassium channel (26). Shortly afterwards, mutations in the calcium channel gene *CACNA1A* were associated with episodic ataxia type 2 (EA2) (26–29). In total, at least nine episodic ataxia syndromes have been described thus far. Genetic associations have been confirmed in EA1 (*KCNA1*), EA2 (*CACNA1A*), EA5 (*CACNB4*, another calcium channel gene), and EA 6 (*SLC1A3*, a glutamate transporter) (26, 29–31). More recently, EA9 was described in association with the *FGF14* gene encoding for a fibroblast growth factor expressed in Purkinje cells and previously associated with spinocerebellar ataxia type 27 (32, 33). In the other episodic ataxias, cytogenetic loci are suspected but unconfirmed or unknown. EA1 and EA2 are the most frequently encountered subtypes and best characterized phenotypes. EA1 has short (seconds to minutes) attacks and interictal myokymia, while EA2 has longer attacks (hours to days) and interictal nystagmus in classic cases. Seizures have been variably reported in association with these genetic conditions, most consistently (but not exclusively) in EA2 and EA6.

While one can state that the genetic era has ushered refinement in disease classification, it is also true that it has blurred boundaries between what appeared to be well-established territories in paroxysmal movement disorders. It became widely demonstrated that a single mutated gene could appear clinically with combinations of different spells, leading to discussions about phenotypic pleiotropy and the accuracy of genotype/phenotype correlations. For example, a patient with a point mutation in *CACNA1A* might have a combination of different spells, including episodic ataxia, hemiplegic migraine, and/or seizures. The same applies to *PRRT2* mutations. What is more, one particular phenotype (for example, episodic ataxia) could be caused by different genes, with often overlapping clinical features (a concept known as allelic heterogeneity) which justifies the ample use of gene panels in neurogenetics today (34). The combination of epilepsy and paroxysmal movement disorders is particularly interesting in this regard. Both phenomena presume transient abnormal neuronal firing leading to dysfunction of neuronal networks. These episodic disorders are generally attributed to confirmed or presumed cell membrane dysfunction, often caused by channel ion gene mutations (35).

## Phenotypical Overlap

For the purposes of this review, we have organized the phenotypes according to their main characteristic features. We find it is a useful construct when applied to patient encounters. However, it is important to recognize though that these phenotypical models (kinesigenic, non-kinesigenic, and exertional dyskinesias) are not clear-cut. Some genetic conditions will exhibit more than one type of trigger in different patients. For example, in patients with PKD associated with *PRRT2* mutations, non-kinesigenic episodes may be present in up to 70% of cases (36). In rare instances, *PRRT2* mutations can also cause episodic ataxia (37). Patients with mutations in *SLC2A1* may have multiple types of episodes often including exercise-induced and non-kinesigenic, and more rarely kinesigenic attacks (10). Kinesigenic triggers, on the other hand, may also cause episodes of ataxia in more than half of cases with EA1 associated with mutations in *KCNA1* (38).

### Paroxysmal Kinesigenic Dyskinesias and Epilepsy

The clinical syndrome of PKD is the more recognizable entity among the paroxysmal movement disorders. The kinesigenic trigger was well-characterized in the 1960s (6) and clinical criteria for diagnosis have been used since the 2000s (39) (Table 2). The criteria include the presence of kinesigenic triggers, short duration of attacks, and positive response to anti-epileptic drugs. In the 1990s, 4 families were described where patients presented in the first year of life with benign familial infantile seizures (BFIS), later developing PKD in the first or second decade. The association of BFIS with PKD was coined infantile convulsion choreoathetosis syndrome (ICCA), and it was mapped to the pericentromeric region of chromosome 16p with autosomal dominant inheritance (40). Combining classic linkage with whole exome sequencing, the PKD syndrome and ICCA were mapped to mutation in the *PRRT2* gene, with autosomal dominant inheritance. Mutations in *PRRT2* were quickly found as the underlying cause of a large proportion of classic PKD cases, and also “pure” cases of BIFS (13, 14, 41, 42). Mutations in *PRRT2* have also been associated with other paroxysmal disorders, such as episodic ataxia, and hemiplegic migraine (37).

**Table 2:** Criteria for Paroxysmal Kinesigenic Dyskinesia [adapted from Bruno et al. (39)].

Age at onset between 1 and 20 years
Identified kinesigenic trigger for the attack
Short duration of the attack (< 1 min)
No loss of consciousness or pain during attacks
Normal neurological examination
Control of attacks with phenytoin or carbamazepine
Lack of an alternative organic or structural explanation for the attacks

Even in those patients with a prior a history of BFIS, the events of PKD appear to be non-epileptiform, and telemetry with EEG is negative for epileptiform discharges during the paroxysmal kinesigenic episodes. Nevertheless, the question remains whether kinesigenic activity in PKD could trigger a form of “basal ganglia seizure,” in which there is synchronous abnormal firing of the structures involved in movement disorders. This lead older authors to debate whether it was as a type of “reflex seizure” (43, 44). A study on a single patient using dural and depth electrodes during a PKD episode did indeed demonstrate focal discharges arising from the contralateral supplementary motor cortex rapidly followed by discharges in the caudate nucleus (45). However, follow-up studies using regular surface EEG electrodes did not demonstrate abnormal ictal activity, with some interictal EEG abnormalities in some patients (46, 47). It seems that the primary functional abnormality during a PKD attack is related to transient basal ganglia hypermetabolism, as demonstrated by PET (48). However, the question remains whether ongoing cortical dysfunction could somehow modulate the expression of the disease. With increased recognition of cortico-basal ganglia connectivity, it is conceivable to explore the idea that abnormalities in one node of the network could lead to symptomatic manifestations arising from other connected areas. Mutations in *SCN8A* exemplify this concept whereby patients with ICCA syndrome may have epileptogenic activity during BFIS and contralateral rhythmic activity in the sensory-motor region during the PKD episodes (49).

Seizures associated with mutations in *PRRT2* most commonly manifest as BFIS, with mean onset at 6 months but ranging between 3 and 20 months of age (50). Seizures are focal in origin with or without secondary generalization, and are generally brief with occasional clusters up to 10 events/day (51). The seizure semiology is varied, comprising psychomotor arrest, eye and/or head deviation, unilateral tonic limb stiffening, or jerking that may generalize. Typically, BFIS responds to AED treatment and improves after age 2. Seizures associated with *PRRT2* mutations can also occur in the context of febrile seizures or febrile seizures plus syndrome (GEFS+) (52, 53).

PKD has a mean age of onset at 12 years of age with attacks consistently triggered by sudden movement, often in the context of emotional, or physiologic stress (54). A sensory aura preceding the attack is commonly reported. Kinesigenic paroxysmal movements in PKD are short and frequent, with more than 90% of patients reporting duration <30 s and more than a third of patients reporting more than 20 attacks per day (39). Interestingly, the short duration and high frequency of daily events also occurs in the seizures that patients with BFIS experience. The combination of BFIS and PKD (ICCA syndrome) is associated with mutations in *PRRT2* in about 80% of cases (55–57), whereas isolated PKD is associated with *PRRT2* mutations in 40–90% of cases. The prevalence is increased in the setting of a positive family history (54, 58).

Mutations in *PRRT2* most often occur in a hotspot where an insertion of an additional cytosine base creates a hairpin loop in the transcript causing DNA polymerase slippage (42, 52). About 80% of patients carry the c.649dupC mutation, causing a frameshift that may lead to unstable RNA or a truncated protein, subject to non-sense mediated decay. *PRRT2* mutations are therefore predicted to be loss-of-function (14, 59, 60). The mechanism by which *PRRT2* haploinsufficiency causes disease is not totally clear, but some translational evidence suggests that it may be related to synaptic dysfunction in glutamatergic neurons, with associated abnormalities in sodium currents (60, 61). Patients with biallelic mutations in *PRRT2* have been reported to have a more severe phenotype with including different seizure types, status epilepticus, episodic ataxia, and PKD (62, 63).

Both BFIS and PKD are exquisitely responsive to antiepileptic drugs, especially low dose carbamazepine and phenytoin (54). Some authors have speculated that the sodium channel blocking properties of these AEDs may be particularly useful, and that carbamazepine also enhances the activity of certain glutamate transporters reducing its concentration on the synaptic cleft (61). Other phenotypes associated with *PRRT2*, such as hemiplegic migraine, respond less consistently to carbamazepine (64).

Other genes and loci have been associated with the PKD syndrome, but *PRRT2* appears to be the most common. Mutations in *SCN8A*, encoding for the alpha subunit of the Na_v_1.6 voltage- gated sodium channel, are notable. Mutations in this locus were originally associated with developmental epileptic encephalopathy, but milder phenotypes exist combining BFIS, PKD, and ICCA syndrome, as well as other non-epileptic paroxysmal phenomena such as tremulousness and hyperekplexia (49, 65). In addition to *SCN8A*, a large study in patients with clinically defined PKD (66) identified mutations in *DEPDC5 (*originally described in the context of focal familial epilepsy with variable foci) as well as *KCNA1, KCNMA1, SLC2A1*, and *PNKD* (the most common phenotypes associated with these genes are discussed further in the manuscript). Finally, PKD has also been reported in association with mutations in the acetylcholine receptor gene *CHRNA4* (in association with epilepsy and developmental delay), and in the thyroid hormone transporter *SLC16A2* (also known as MCT8) (67–69). In the latter case, a remarkable clinical feature is that paroxysmal dyskinesias may be triggered in the context of passive manipulation of a limb.

### Paroxysmal Non-Kinesigenic Dyskinesia and Epilepsy

Paroxysmal non-kinesigenic dyskinesia probably encompasses the largest group of paroxysmal disorders with overlapping epileptic features. The familial form of PNKD was originally described by Mount and Reback (5), under the term “paroxysmal familial choreoathetosis” describing a syndrome with autosomal dominant inheritance, with spells triggered by alcohol, caffeine, or fatigue. Through linkage mapping and sequencing studies, mutations in the gene *MR-1* (currently called *PNKD*) were identified (11). Notably, mutations in *PNKD* appear to influence synapse formation and neurotransmitter release (61, 70), may have co-morbid migraine, but have not been implicated in epilepsy.

Paroxysmal non-kinesigenic dyskinesia may be familial or sporadic. As outlined above, familial cases are most commonly associated with mutations in *PNKD* and have a well-defined phenotype (Table 3) (71). However, many other genetic conditions may underlie familial cases; and acquired causes can also lead to non-kinesigenic dyskinetic episodes (72). These other causes may be called “atypical PNKD” (71). Acquired forms can be secondary to various injuries to the basal ganglia (73). Mutations in *KCNMA1*, a gene encoding a subunit of a potassium channel activated by calcium, resemble the classic PNKD phenotype, with associated epilepsy and developmental delay (74, 75). Although this disorder appears to be quite rare and few cases have been reported, it is worth noting that some patients may experience improvement in paroxysmal episodes with stimulants (76).

**Table 3:** Criteria for Paroxysmal Non-Kinesigenic Dyskinesia associated with *PNKD* [adapted from Bruno et al. (71)].

Onset of attacks in infancy or early childhood
Attacks triggred by caffeine or alcohol consumption
Duration of attacks 10 min to 4 h (typically < 1 h)
Family history of a movement disorder with the previous characteristics

Perhaps the best prototypical example of non-kinesigenic dyskinesia associated with ample phenotypic variability is related to monoallelic mutations in the gene *ATP1A3*. The gene encodes for a subunit of the sodium-potassium (Na+/K+) ATPase responsible for establishing and maintaining the electrochemical gradient of sodium and potassium ions across neuronal plasma membranes. Mutations in this gene were originally associated with rapid-onset dystonia-parkinsonism (RDP) (77), and later were attributed to be the major cause of alternating hemiplegia of childhood (AHC), a condition which causes both motor symptoms, progressive cognitive and motor deficits, and often epilepsy (78). Although AHC is named after the episodes of hemiplegia, dyskinetic movements were noted since its original description (79). Paroxysmal dystonia is frequently reported, in addition to nystagmus, autonomic changes, and reduced awareness (80–82). In fact, paroxysmal dystonia that involves the upper extremity with flexion or extension and elevation of the limb may be a distinguishing feature (83). Although “classic” RDP does not typically present with paroxysmal dyskinesia, there is a phenotypic spectrum on the clinical expressivity of *ATP1A3* mutations, with some patients exhibiting intermediary phenotypes.

The expanding spectrum of *ATP1A3* disease includes patients with features of both AHC and RDP having partial features of each or fulfilling the diagnostic criteria of both; (84, 85) or patients who share features with RDP or AHC but with distinct characteristics of the D-DEMØ phenotype - Dystonia, Dysmorphism of the face, Encephalopathy with developmental delay, MRI abnormalities of the brain always including cerebellar hypoplasia, No hemiplegia (Ø), and neonatal onset (86). Finally, patients with mutations in *ATP1A3* may also have CAPOS syndrome (cerebellar ataxia, areflexia, pes cavus, optic atrophy syndrome, and sensorineural hearing loss), isolated ataxia or other varied phenotypes (85, 87, 88).

The clinical characteristics of paroxysmal dystonia in AHC are of particular interest: [1] It is often precipitated by stress, hot, or cold water exposure; [2] Onset usually occurs before age 18 months (including first day of life), although later onset is possible; [3] Within the same spell, patients often have dystonia of one or more limbs/trunk with concurrent hemiplegia in other limbs, the limb can start with hemiplegia and then develop dystonia or vice versa. Dystonic and hemiplegic attacks can frequently end with an epileptic seizure or directly follow an epileptic seizure. This often represents a diagnostic challenge that can be addressed only with video EEG monitoring during an acute event to rule out ongoing electrographic seizure activity. It is important to detect such activity early on during an acute AHC event in which the distinction cannot be clearly made. This is because such events may evolve into status epilepticus, convulsive, or non-convulsive, which has the risk of developmental regression (82, 89, 90). In some patients, the hemiplegic component is more important in early childhood than later, with the opposite happening for the dystonia (89, 91, 92).

Patients with AHC may have associated motor impairments, mostly in oromotor functioning leading to dysphagia, followed by fine motor and gross motor impairments. The swallowing problems are at times, but not always, due to oral-pharyngeal dystonia (90). Associated tone abnormalities in-between AHC attacks are also seen. More often, individuals present with decreased tone, but a minority may be spastic or continuously dystonic. As the patient gets older, there is often a reduction of hypotonia and of abnormal ocular movements (93).

Paroxysmal dystonia can occur in AHC irrespective of underlying mutation in *ATP1A3*, including the three most common mutations (D801N, E815K, and G947R). The first two mutations are associated with more severe epileptic and developmental manifestations, dominating the clinical picture such that dystonia is often, but not always, less of a problem than the other manifestations. In individuals with the G947R mutation which has a less severe phenotype, dystonia often appears to be more noticeable and is more commonly reported in comparison to the other mutations (94, 95).

The type and severity of epilepsy in *ATP1A3*-related disease can vary greatly. It ranges from neonatal onset, as in the D-DEMØ and similar syndromes, to adulthood in patients with RDP (86, 96). The age of seizure onset is best studied in AHC, with an average of 1.83 ± 3.00 years (80).

There is evidence that more severe phenotypes are associated with mutations affecting the transmembrane and functional domains of the protein (97). Some early authors pointed out to the idea that mutations that reduced protein expressivity were more likely to cause RDP, whereas those associated with AHC did not (78). Albeit imperfect, this correlation suggested that the mechanism of disease occurred through hypomorphic effects on RDP whereas those associated with AHC did so through modulating the activity of the Na+/K+ ATPase pump.

**Figure 1:**
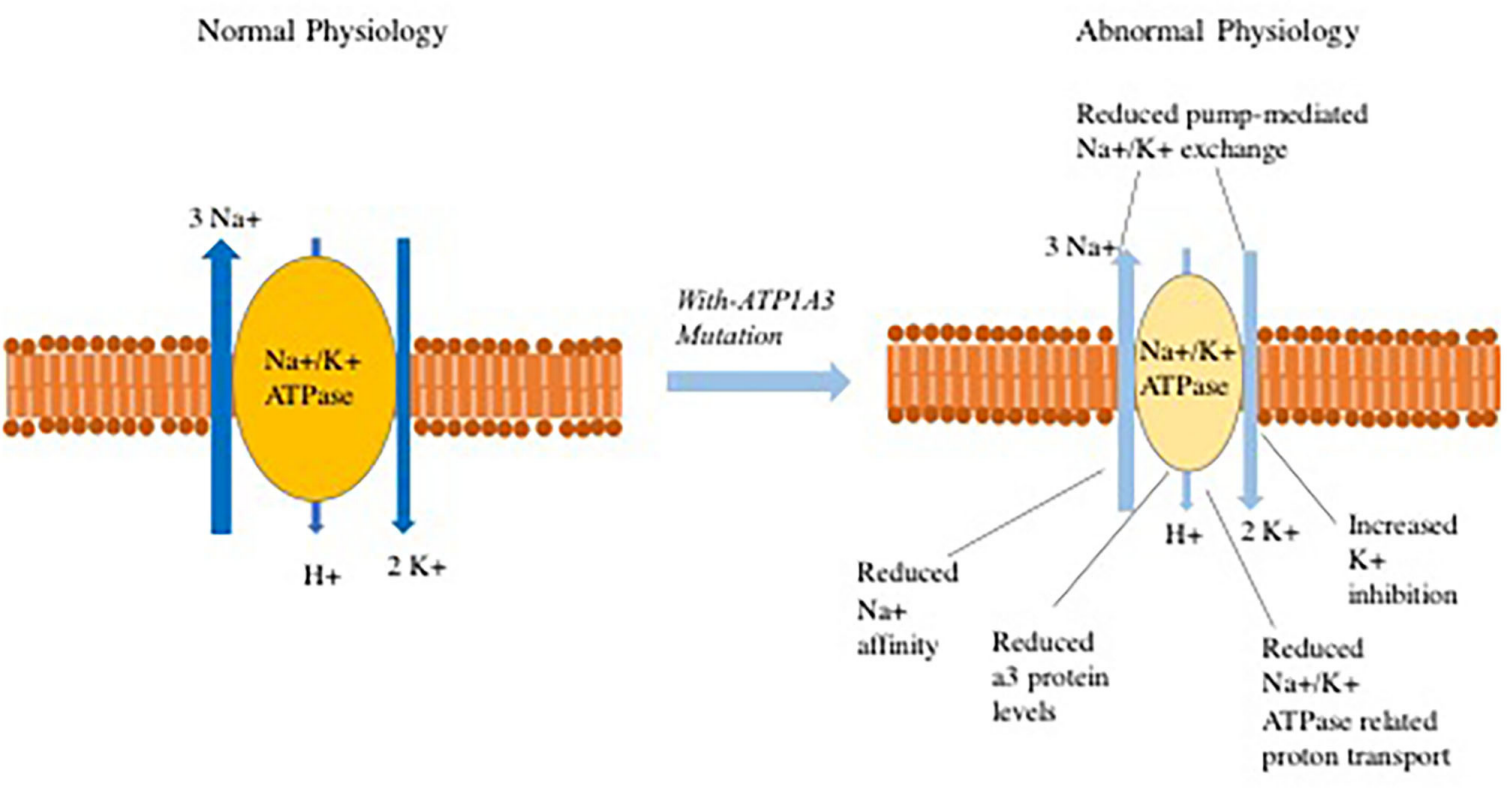
Abnormalities that have been observed in Na^+^/K^+^-ATPase related ion transport secondary to ATP1A3 mutations.

One potential mechanism of disease in individuals with *ATP1A3* mutations is that dysfunction of the Na+/K+ ATPase pump results in depolarization that leads to abnormal Na^+^ and K^+^ exchange process and disturbed proton transport (Figure 1), These abnormalities can potentially lead to subsequent opening of voltage-gated calcium channels. The increased intracellular Ca2+ concentration may then cause downstream activation of multiple signal transduction pathways (98–101). In reality, the pathophysiology is more complex with lines of evidence pointing to distinct mechanisms through which *ATP1A3* mutations perturb normal physiology. Some examples include:

- Increased excitability of cortical and hippocampal neurons and impairment of firing of parvalbumin-positive fast-spiking interneurons, likely leading to epilepsy as demonstrated in the Mashlool mouse carrying the most common mutation causing AHC (D801N) (102).
- Impaired trafficking of mutant αβ complex to Golgi apparatus and plasma membrane. This was demonstrated in HEK-293 cells expressing *ATP1A3* mutations associated with a severe phenotype of microcephaly, developmental delay, and dystonia, indicating competition between exogenous mutant *ATP1A3* (α3) and endogenous *ATP1A1* (α1) so that their sum was constant (103).
- Increased predisposition to cortical spreading depolarization (depression), a known mechanism for hemiplegic migraine, as demonstrated in the Mashlool mouse (104). Notably, spreading depolarization exerts a modulatory effect on the function of the basal ganglia (105–107). In addition, spreading depression can occur in the basal ganglia and more directly lead to dysfunction of that system (108). Thus, spreading depolarization may be a mechanism for dystonia too.
- Irregular firing of Purkinje cells and deep cerebellar nuclei neurons, resulting in dystonia as demonstrated in mice carrying the D801Y mutation (109). A few lines of evidence support the role of the cerebellum in *ATP1A3-* related disease. Cerebellar hypoplasia is seen in all patients with the D-DEMØ phenotype (86). Cerebellar atrophy and hypometabolism on PET scanning are noted in AHC patients (110, 111).

Paroxysmal dystonia can also occur in patients who have *ATP1A2*-related severe epileptic encephalopathy. For example, in a recent report of 6 such patients, 3 of them had dystonic spells in the context of exacerbation. These paroxysmal attack may include acute encephalopathy, hemiplegia, dystonia, and seizures with a duration that may span hours to days (112). There, the mechanism is presumed to be dysfunction of the glial ATPase (ATP1A2 I glial) that impairs re-uptake of glutamate which predisposes to spreading depolarization and increased excitability.

Other genetic causes of the PNKD phenotype frequently co-occur with epilepsy, such as the above cited PKD (e.g., *PRRT2*) or PED due to *SLC2A1 mutations* (10). Another disorder associated with paroxysmal non-kinesigenic dyskinesia and seizures is associated with mutations in the *GNAO1* gene. The gene product encodes for a subunit of the heterotrimeric structure associated with G protein intracellular signaling, and was initially described in association with epileptic encephalopathy (particularly Ohtahara syndrome), developmental delay, and interictal dyskinetic movements (113). The baseline movement disorder may worsen in association with several physiological triggers (infection, stress, gastrointestinal issues, others) and can be life threatening. Although generalized, the dyskinetic movements may be particularly prominent in the orolingual region (114, 115). Both baseline dyskinetic movements and the paroxysmal exacerbations may respond to deep brain stimulation, although the optimal programming parameters are not yet defined (116). Additional genes in which paroxysmal dyskinetic events have been variably associated with epilepsy and/or an abnormal include *SYT1* and *RHOBTB2* (117–119).

Lastly, there is increasing recognition of the presence of movement disorders as a defining feature in some epileptic encephalopathies, often referred to as epilepsy-dyskinesia syndromes. In many of these disorders, the movement disorder is present without paroxysmal exacerbations, although the clinical features and/or severity may change over time. Furthermore, it may be difficult sometimes to discern whether an abrupt worsening of the underlying movements is due to their paroxysmal nature vs. worsening due to external factors (e.g., metabolic derangement, infections, etc.). Notable disorders to consider in this scenario include inborn errors of metabolism (neurotransmitter deficiencies, cerebral folate deficiency, certain lysosomal storage disorders, mitochondrial disorders, congenital disorders of glycosylation) and an extensive list of complex monoallelic disorders (e.g., mutations in *SCN1A, SCN2A, KCNT1, FOXG1, ARX, GRIN1, FRSS1L, STXBP1*, and many others). Reviewing all the epilepsy-dyskinesia syndromes is outside the spectrum of this review; the reader is directed to excellent reviews on this emerging topic (120, 121).

### Paroxysmal Exertional Dyskinesia and Epilepsy

Paroxysmal exercise induced dyskinesias (PED) were first reported in the pre-genetic era, describing patients that would have attacks of dystonic posturing precipitated after a variable period of exercise, usually lasting 5–30 min (8). Although the lower limb seems to be most commonly involved, it may be more related to a function of the body part that has been most active in exercise. Upper extremity symptoms have also been reported (122).

An important cause of PED syndrome is related to mutations in the *SCL2A1* gene, encoding for the facilitated glucose transporter type 1. The so-called “Glut 1 deficiency,” a syndrome comprising hypoglycorrhachia, seizures, acquired microcephaly, and developmental delay was first described in 1991 (123). *De Vivo* and colleagues first hypothesized that the fundamental defect was a genetic defect in the glucose transporter in the endothelial cells lining the blood brain barrier and erythrocytes. Treatment with the ketogenic diet, providing the brain with a means of alternative energy utilization, was remarkably successful in improving symptoms. The molecular basis for the disease, associated with monoallelic mutations in *SLC2A1* was indeed confirmed a few years later (124).

Patients with mutations in *SLC2A1* may have a combination of neurodevelopmental disabilities (e.g., intellectual disability, developmental delay, attentional issues), epilepsy, and movement disorders (spasticity, hypotonia, ataxia, dystonia, and chorea) (125). Most patients exhibit a combination of these features, but mildly affected individuals have also been described. In addition to seizures, several other paroxysmal events may occur in patients with *SLC2A1* mutations. Besides PED, these include other paroxysmal movement disorders, paroxysmal head-eye movements in the first year of life, migraines, vomiting, hemiplegic attacks, and lethargic spells. Indeed, *SCL2A1* mutations should be placed high in the differential diagnosis for most paroxysmal neurological symptoms with onset in infancy, as well as considered as a possibility in later-onset cases (126–131). Since *SLC2A1* mutations cause a highly pleomorphic disease, the neurodevelopmental aspects, epilepsy, and chronic motor disturbances (spasticity, dystonia, or ataxia), may overshadow milder manifestations like isolated PED—likely to be underreported both in the literature and clinical practice. One report of 22 GLUT1 deficiency patients with confirmed *SLC2A1* mutations showed that at least half presented with PED, suggesting that as a feature, it is much more common than as an isolated condition at least in *SLC2A1*-related disease (131). In this report, the age of onset of PED events ranged from 8 months through 14 years old, with mean onset close to 6.5 years of age.

In the majority of patients, seizures are present before age 2 and are diverse in semiology. Complex absences, focal seizures, and cyanotic spells can be seen early on, with myoclonic seizures and generalized tonic-clonic seizures observed later (126, 132). The majority of patients have more than one seizure type, and the most common ones appear to be focal and atypical absences (125, 132). In particular, suspicion for *SLC2A1* mutations should be raised when there is a combination of atypical features in absence epilepsy, including early age of onset, intellectual disability, treatment refractoriness, other seizure types, or family history (133, 134).

PED is notable in the sense that it is more likely to be a feature which can compose the phenotypic spectrum of different conditions. This is in contrast to PKD and familial PNKD, in which one gene (*PRRT2* for PKD and *PNKD* for familial cases) account for the majority of cases. In keeping with the pathophysiological construct of metabolic deficiency leading to the manifestations of *SLC2A1* mutations, other genes associated with brain energy utilization have also been reported combining seizures and PED. In particular, these include mutations in *ECHS1* and pyruvate dehydrogenase deficiency complex, sometimes in the context of lactic acidosis and/or developmental encephalopathy (135, 136). Nevertheless, alternative pathophysiological explanations are necessary to explain other cases of PED. Well-characterized cases of PED have been associated with dopa-responsive dystonia caused by mutations in *GCH1* (137) and levodopa-responsive young onset parkinsonism caused by *PARKIN* mutations (138, 139). Two cases of response to levodopa in PED caused by *SLC2A1* mutations (9, 140) further add to the unexpected link between these dopaminergic deficiency and PED. PED episodes associated with epilepsy can also happen in *ATP1A3*-related disease (in addition to chronic dystonia and parkinsonism) as well as in mutations in *TBC1D24* (141–143). In clinical practice, many other cases of PED remain with undetermined etiology.

### Episodic Ataxia Type 1 and Epilepsy

What is known today as Episodic Ataxia type 1 (EA1) was first identified by Van Dyke and colleagues in 1975 as hereditary myokymia and episodic ataxia (144). Almost twenty years later, autosomal dominant mutations in the *KCNA1* gene were found to be responsible for the disease (26). It encodes for the α-subunit of the Kv1.1 voltage-gated potassium channel, widely expressed in the central and peripheral nervous system, including the hippocampus and cerebellum (145).

The characteristic EA1 patient will present with recurrent ataxic episodes. These episodes may be frequent, short-lasting, and often begin in the first decade of life; genetically confirmed cases often have a positive family history (146). Interictal myokymia is a hallmark of the disease, but may not always be present. Events can be triggered by kinesigenic movement, exertion, or emotional distress (38). Episodes may fade with age, but some patients may have persistent cerebellar ataxia (144, 146). A positive clinical response to acetazolamide and AEDs (mainly carbamazepine) has been reported, however no single drug appears to have widespread efficacy (147).

In addition to spells of episodic ataxia, patients harboring mutations in *KCNA1* may also have seizures (146). Epilepsy may present with partial and/or generalized seizures. There are too few cases reported in the literature to establish a particular pattern, but the age of onset thus far reported appears to be variable, occurring in infants through childhood, and adolescence; sometimes preceding episodic ataxia (148–150). *KCNA1* mutations associated with epilepsy tend to cluster in the S1/S2 helices and pore domain of the channel (145). A subset of individuals with *KCNA1* mutations may have severe forms of epilepsy, where identification of episodic ataxic symptoms may prove more difficult, including a small number of children harboring *KCNA1* mutations with epileptic encephalopathy (151, 152). One patient with a variant in *KCNA1* had severe myoclonic epilepsy and sudden unexpected death, although it is considered whether polymorphisms in other genes could have contributed to the phenotype (153).

Mutations in *KCNA1* have been associated with other phenotypes, including paroxysmal dyskinesia (56), isolated neuromyotonia (148), or myokymia (154), and hypomagnesemia (155).

### Episodic Ataxia Type 2 and Epilepsy

Episodic Ataxia type 2 is probably the most prevalent amongst the episodic ataxias. The prototypical EA2 phenotype begins in infancy or early childhood with recurrent spells of unsteadiness, vertigo, and slurring of speech (34). Attacks may be triggered by exertion, fatigue or emotional arousal, and patients may exhibit interictal progressive ataxia, nystagmus, and cerebellar atrophy on imaging (34, 156, 157). EA2 is caused by autosomal dominant mutations in the *CACNA1A* gene, which encodes the alpha1-subunit of the Ca_v_2.1 P/Q-type voltage gated calcium channel, widely expressed in a variety of cerebral and spinal neuronal populations (157). Mutations in this gene have been associated with different allelic disorders with autosomal dominant inheritance, albeit with phenotypic overlap: familial hemiplegic migraine, EA2, spinocerebellar ataxia type 6 (SCA6), epileptic encephalopathy, seizures, neurodevelopmental disorders, and non-progressive chronic ataxia (156–160). Because SCA6 is caused by repeat expansions of a polyglutamine tract in the *CACNA1A* gene, it is considered mostly as a separate, allelically related disorder from EA2. Nevertheless, up to a 1/3 of patients with SCA6 may also have episodic features (161, 162). Conversely, chronic progressive ataxia may occur in over 50% of patients with EA2 (163).

A proportion of individuals with *CACNA1A* mutations may have epilepsy, manifesting with febrile seizures, absence seizures, refractory tonic-clonic seizures, or multiple seizure types associated with epileptic encephalopathy (157, 158, 160, 164–166). Seizure onset in infancy and childhood is commonly reported in the literature, but it is difficult to determine if this is a publication bias vs. a notable clinical feature. The onset of *CACNA1A*-associated disease spectrum symptoms spans from infancy to adulthood (166). In some mutation carriers (in particular the S218L mutation), minor head trauma may lead to severe hemiplegic migraines associated with lethargy, seizures and brain edema (167). Emerging evidence suggests that mutations in *CACNA1A* may also be associated with neurodevelopmental disorders such as intellectual disability, learning disabilities, or autism (157, 168, 169).

Therapeutically, the episodes of ataxia in patients with *CACNA1A* mutations often respond well to acetazolamide (163). The pleiotropic nature of *CACNA1A* may give rise to other phenotypic characteristics, including migraines, and hemiplegic attacks, sometimes with overlapping features in a single individual (163, 170).

### Episodic Ataxia Type 5 and Epilepsy

EA5 is caused by mutations in the voltage-gated calcium channel encoded by the gene *CACNB4* (30, 171, 172). Since there are only a few kindreds described, it is difficult to draw conclusions about the phenotypic spectrum. It appears that in the heterozygous state mutations in *CACNB4* lead to autosomal dominant EA5 and/or predisposition to epilepsy (particularly myoclonic and generalized types), but larger-scale human studies have not conclusively confirmed the latter (172). In the homozygous state, mutations appear cause a more severe neurodevelopmental disorder (171).

### Episodic Ataxia Type 6 and Epilepsy

Episodic ataxia type 6 is caused by monoallelic mutations in *SLC1A3*, encoding for the excitatory amino acid transporter 1 (EAAT1) (31). This high-affinity sodium-dependent transporter is involved in regulating neurotransmitter concentrations in glutamatergic synapses and is expressed in the cerebellum, diencephalon, and brainstem. From the original description of a young boy with episodic ataxia, seizures, migraine, and alternating hemiplegia (31), the phenotype soon expanded to include individuals with EA2-like phenotypes or late-onset episodic ataxia, without epilepsy (173–175). Experimentally, distinct consequences at the functional and/or expressional level of EAAT1 have been found with different mutations (176), rendering genotype-phenotype correlations unconfirmed in this rare condition.

### Other Disorders Variably Associated With Episodic Ataxias and Epilepsy

Episodic ataxia with epilepsy has also been reported in association with mutations in a few other genes, frequently encoding for ion channels. The *SCN2A* gene, encoding for a voltage-gated sodium channel, has a reported phenotype ranging from epileptic encephalopathy to benign familial neonatal infantile seizures, hyperkinetic movement disorders (chorea, dystonia and myoclonic jerks), neurodevelopmental disorders, and episodic ataxia, which may occasionally respond to acetazolamide (177–180). The *KCNA2* gene belongs to the Kv_1_ family of voltage-gated potassium channels. Pathogenic mutations have been described in the context autosomal dominant epileptic encephalopathy and chronic ataxia (181–184). An episodic nature to the ataxia was described, with some patients having variable associations between attacks and infantile-onset seizures (185).

## Therapeutic Overlap

Not surprisingly in light of the significant overlap amongst these conditions, drugs with anti-epileptic properties are frequently used to treat most paroxysmal movement disorders (186, 187).

Phenytoin, an anti-epileptic agent with blocking activity of voltage-gated Na+ channels, was possibly the first drug tried in paroxysmal movement disorders. It was initially reported as a failed therapy in a case of PNKD (5), but then noted to be helpful in a case of so-called reflex epilepsy (which, in retrospect, presumably reflected a case of PKD) (188). With the recognition of kinesigenic triggers, treatment with phenytoin was consolidated as the preferred treatment of the PKD syndrome in the 1960s (6).

Carbamazepine, another AED with Na+ channel blocking activity, debunked phenytoin as the treatment of choice of the PKD syndrome, probably because of its efficacy and improved side effect profile. It was first reported to be efficacious more than 50 years ago (189) and to this day remains the first-line treatment of PKD (190). Carbamazepine responsiveness is a diagnostic criterion for the disease (39). More than 98% of patients with known *PRRT2* mutations may respond favorably to the drug (54), and it may also be useful in other non-*PRRT2* or acquired cases of PKD (73).

Acetazolamide, a diuretic, is a carbonic anhydrase inhibitor. It blocks the breakdown of carbonic acid, thus causing acidosis. It had been known since the 1920s that starvation (causing ketoacidosis) could improve epilepsy (191). Therefore, when the drug became available it was used to treat epilepsy in the 1950s on the grounds that it could provide a similar effect (192). Initial reports of acetazolamide use were encouraging, but it soon became clear that it had limited efficacy in treating epilepsy (193). It is possible that it may be more efficient in pediatric than adult cases (194), nevertheless its use as an AED has largely been discontinued in favor of other newer and more tolerable agents. Griggs et al. (23) were the first to report successful treatment of familial episodic ataxia (then called paroxysmal ataxia) with acetazolamide. This finding was later reproduced by various other colleagues and proven to be the hallmark of episodic ataxia type 2, dubbed “acetazolamide-responsive hereditary paroxysmal ataxia” in pre-genomic era papers.

The ketogenic diet can improve both epilepsy and PED episodes in *SLC2A1* mutation carriers (195), and remains a therapeutic option for refractory epilepsy of various causes (196). Triheptanoin, an odd-chain triglyceride able to produce C5-ketone bodies and provide intermediates to the Krebs cycle, was shown to improve 90% of non-epileptic paroxysmal manifestations in a study of 8 patients with *SLC2A1* mutations, including paroxysmal exercise-induced dyskinesia and non-kinesigenic dyskinesia (197). Data on the response of the epileptic attacks is lacking.

Benzodiazepines (mostly clonazepam and diazepam) can be very effective as prophylactic or rescue treatment in genetically confirmed *PNKD* patients (71, 190), but in other causes of the PNKD syndrome their use may be limited by efficacy or intolerable side effects. Some patients with paroxysmal events associated with mutations in *SLC2A1* may also benefit from benzodiazepines (190). Other AEDs, including topiramate, levetiracetam, phenobarbital, gabapentin, valproic acid, lamotrigine have all been anecdotally reported to be useful in PKD (190).

Although not in the category of anti-epileptic agents, two other drugs are worth mentioning. Levodopa has been reported to be efficacious in rare cases of PKD (198) and most consistently in PED associated with mutations in *SLC2A1, GCH1*, or *Parkin* (137, 140, 199). 4 -aminopyridine, a potassium channel blocker, was shown in a small trial to reduce the frequency of episodes and improve quality of life in patients episodic ataxia type 2 (200). It may be an option for patients who cannot tolerate acetazolamide.

Finally, despite their therapeutic usefulness, it is worth mentioning that AEDs can also be associated with drug-induced movement disorders (201–204). In most instances, the movement disorders are not paroxysmal and there is a temporal relationship to initiation of a drug, but exceptions exist. The movement disorder may occur at any time during treatment and within therapeutic serum levels. Patient and drug-specific factors may influence symptoms, including pregnancy and drug dosage changes (205–207). Many AEDs are implicated. The most frequent offenders are the older agents (205), including phenytoin (206), carbamazepine (208), and valproate (209). Newer ones such as lamotrigine, levetiracetam, and gabapentin have also been implicated (210–212). In some cases, the offending agent may be toxic and have lasting effects. For example, phenytoin may lead to cerebellar atrophy and ataxia that persists even after the drug is discontinued (213).

Tremor is a frequent abnormal movement reported in association with AEDs, accounting for 45% (89/201) of cases (214, 215). Chorea has been associated with phenytoin and valproate (216–218). Combined use of multiple AEDs may increase this risk (219). Usually seen as an anti-dyskinetic drug to treat chorea, valproate may also induce parkinsonism (209, 220), Ataxia may be induced by phenytoin, carbamazepine, valproate, and lamotrigine (207).

## Conclusion

We have attempted to provide a comprehensive overview of select paroxysmal movement disorders and their associations with epilepsy. These distinct phenotypes are coalescing into established genetic etiologies with extensive pleiotropy. Several good diagnostic algorithms exist in the literature (1, 72, 221). We have refrained from designing a diagnostic algorithm for these conditions, as we believe that careful phenotyping at the bedside is just as important for diagnosis as it is for interpreting results from hypothesis-free (agnostic) genetic testing. Moreover, familiarity with paroxysmal movement disorder subtypes may allow clinicians to suspect specific genetic etiologies that, if not identified through next-generation sequencing, may be pursued through other means such as evaluating for nucleotide expansions or copy number variants (221, 222). For several disorders, although genetic causes have been identified, the molecular pathophysiology is still largely unknown. In the near future, it is likely that we will have a growing number of genes associated with movement disorders and epilepsy, in parallel with improved understanding of disease mechanisms leading to more effective treatments.

## Author Contributions

CG: original idea, literature review, and first draft. LS-M: analysis and criticism of the first draft and senior input. SS: figure generation and provision of original data. MM: figure generation, provision of original data, analysis and criticism of the first draft and senior input. LG: literature review, data tabulation, and data management.

## Conflict of Interest

The authors declare that the research was conducted in the absence of any commercial or financial relationships that could be construed as a potential conflict of interest.
